# Profiles of positive changes in life outcomes over the COVID-19 pandemic in Chinese adolescents: the role of resilience and mental health consequence

**DOI:** 10.1186/s13034-022-00451-4

**Published:** 2022-02-22

**Authors:** Jian-Bin Li, Kai Dou, Zi-Hao Liu

**Affiliations:** 1 Department of Early Childhood Education, The Education University of Hong Kong, Hong Kong, SAR China; 2 Research Center of Adolescent Psychology and Behavior, School of Education, Guangzhou University, 230, Waihuan Road West, Panyu District, Guangzhou, People’s Republic of China

**Keywords:** Positive psychology, Building effect, Life outcomes, Positive youth development, COVID-19, Pandemic, Latent profile analysis

## Abstract

**Background:**

The 2019 coronavirus (COVID-19) has caused enormous negative impacts on adolescents’ routines, social interaction, interpersonal relationships, psychosocial well-being, and physical health. Nevertheless, theories suggest that individuals also often seek out solutions that may facilitate positive changes when they are faced with uncertainty and crisis. However, the existing literature has disproportionately focused on the negative effect of COVID-19 on adolescents, and scant research has examined to what extent and in what aspects adolescents would experience positive changes in times of the pandemic. This pre-registered research aims to bridge said gaps by: (1) exploring different profiles of positive changes in various life outcomes in Chinese adolescents over the COVID-19 pandemic; (2) examining the role of resilience in differentiating different profiles; (3) comparing adolescents’ mental health across profiles.

**Method:**

Participants were 2,567 adolescents aged 12 to 24 recruited from 32 provinces in mainland China (66.89% females; M_age_ = 19.87 years, SD = 2.02). Through an online survey, participants rated how much their lives of different domains had experienced positive changes since the outbreak of the pandemic. They also answered standardized questionnaires that measured their resilience and mental health.

**Results:**

Results of latent profile analysis revealed three profiles: *limited positive changes* (33.3%), *partial positive changes* (49.5%), and *overall strong positive changes* (17.2%). Moreover, adolescents with a higher level of resilience were more likely to be categorized into the *partial positive changes* profile compared to the *limited positive changes* profile and categorized into the *overall strong positive changes* profile compared to the other two profiles, after controlling for multiple covariates. Adolescents in the *overall strong positive changes* profile had better mental health than their counterparts of the other two profiles.

**Conclusion:**

Chinese adolescents appear to experience positive changes in various life outcomes during the COVID-19 pandemic, especially for those with high levels of resilience. Such positive changes have important implications on adolescents’ mental health.

The COVID-19 has already lasted for two years since the first case was reported, heavily affecting people’s routines, mental health, and well-being worldwide [1]. Governments have been implementing strict measures against the spread of the coronavirus, such as lockdowns, studying/working from home, physical distancing, restraining social interactions, and quarantine. Although these measures are effective, they have also caused substantial side effects, such as inducing isolation and increasing loneliness and worries [2]. Adolescents, the population aged 10 to 24 [3],^1^ are considered particularly vulnerable to the social effects of the aforementioned measures, as the measures have disrupted adolescents’ life structure and led to many negative life outcomes [4]. Adolescence is a developmental period marked by rapid physical and social-emotional changes, onset of many mental disorders, and increased time investment with peers [5, 6]. Research has well documented the negative effects of COVID-19 on adolescents’ psychosocial outcomes, such as heightened mental health problems, increased screen time, more alcohol use, reduced well-being, and decreased physical activity [7–10]. Meanwhile, some studies also unexpectedly found that adolescents reported positive changes in some life outcomes, such as more time to sleep and increased psychosomatic health [8, 9]. These findings lead to intriguing, yet largely underexplored, questions: to what extent adolescents would experience positive changes in life outcomes in times of the COVID-19 pandemic? If they do, who are more likely to have such experience and what are its impacts on mental health? Understanding these questions is paramount, as the findings would shed light on how we can foster positive youth development during COVID-19. However, the existing literature has disproportionally examined the negative impacts of COVID-19 on adolescent development and scant research has focused on the positive side. This study aims to bridge these gaps.

Some theoretical frameworks shed light on the possibility that individuals may show positive changes in times of the COVID-19 pandemic. According to the two continua model of mental health [11], mental distress and mental health co-exist on one continuum, with one polar indicating the presence/absence of mental illness (e.g., anxiety, loneliness) and the other continuum indicating the presence/absence of mental health (e.g., well-being). For instance, Li et al.’s research found that Chinese population used both negative words related to anxiety and depression and positive words related to happiness and life satisfaction on the internet immediately before and soon after COVID-19 occurred [12]. Second, positive psychology proposes that when individuals encounter uncertainty and adversity, they often seek positive solutions [13]. When individuals are able to use the crisis in a transformative way to develop new practices, new processes, and new outlooks, there comes a *building effect* which fosters personal growth and helps individuals improve mental health [14]. For instance, Waters and colleagues found that positive interpretation used by adolescents during COVID-19 predicted levels of growth as this practice was an important coping process that *builds* and *expands* personal growth [15]. These frameworks together suggest that research should not only examine the presence/absence of young people’s mental distress, but it is also time to investigate the other continuum such as experiences of positive changes during the COVID-19 pandemic.

Although research on young people’s positive changes in life outcomes has been surprisingly limited, a few relevant studies are informative. For instance, a narrative review postulated that successfully navigating the challenges of COVID-19 would strengthen adolescents’ sense of community, family cohesion, and personal growth and mental health [4]. The review further considered that young people’s ability to cope with the pandemic is determined by demographic, social (e.g., social support), and individual (e.g., ability to make meaning from the crisis) factors. One quantitative research examined positive changes in life outcomes in Scottish adults during the initial lockdown [16]. It found that participants reported positive changes in various life outcomes, with higher levels in some aspects (e.g., more appreciative of things usually taken for granted) than in others (e.g., spent less time on screen or devices outside of work hours). The results also found that females, younger people, and individuals with better health reported higher levels of positive changes. Fioretti and colleagues (2020) conducted a qualitative study in over 2700 Italian adolescents asking them to complete narrative tasks during the first week of April 2020 [17]. Besides disclosing four negative themes, they also found four themes related to positive experiences, including “being part of the extraordinary experience”, “discovering oneself”, “sharing life at a distance” and “re-discovering family”. These preliminary studies are informative, but they either did not focus specifically on adolescents or did not quantify the positive changes. Moreover, whether adolescents in other countries (e.g., China) experience the similar changes remains largely unknown. Further investigation into this issue is necessary as adolescents’ experience of the changes in life outcomes is associated with the evolvement of COVID-19 which varies greatly in different countries.

While Chinese adolescents likely experience similar changes in many outcomes as found in young people from other countries (e.g., routines, screen time), they may also face some unique changes in certain aspects (e.g., patriotism). China was the first country announcing lockdowns at the beginning of COVID-19 (i.e., from late January to mid-April 2020). Nevertheless, people’s lives have gradually, yet fully, returned to normality since summer 2020 owing to concerted efforts ranging from governments’ rapid response and effective measures to citizens’ compliance and solidarity. Since then, government of different levels in mainland China has been continuously implementing stringent measures nationwide and the pandemic has been under well control over time, despite a few regional recurrences. Along the course of fighting against the COVID-19 pandemic, social media in China (e.g., Douyin, Weibo, and Wechat) played a key role in distributing instant messages, reporting news and policies about the pandemic, and more importantly, conveying ‘positive energy’ (*zheng neng liang*) [18]. As a popular social byword adopted in everyday Chinese political discourse on social media, *positive energy* is defined as ‘the capacity to induce positive emotions and/or attitudes, the potential to induce constructive/conciliatory discourses and/or actions, in individuals or collectives such as the society and nation. Those positive emotions/attitudes/thoughts so induced are also simply referred to as positive energy, as is any event/discourse that is said to contain positive energy” [19]. Positive energy affirms and propagates patriotism, nationalism, and socialism with the Chinese characteristics and therefore it has been widely and frequently promoted on social media [19]. During the anti-pandemic period, positive energy was particularly prevalent on social media in China, potentially influencing Chinese adolescents to become more appreciative, respectful, grateful, and patriotic [18]. Given such special circumstance in the Chinese context, it would be necessary for us to examine the extent to which Chinese adolescents would experience positive changes in the aforesaid life outcomes as well. In addition, because people may show positive changes in multiple aspects of life outcomes [16, 17], person-centered approach with multiple indicators is considered an appropriate method to capture the complex patterns [20].

Going beyond exploring the extent to which Chinese adolescents would experience positive changes in various life outcomes, we are also interested in examining the role of resilience and the impact of such changes on mental health outcomes. Resilience refers to the capacity of a system to adapt successfully to significant challenges that threaten its function, viability, or development [21]. Prior studies consistently found that resilience, as a crucial character strength, is an important asset that helps young people cope with COVID-19, bounce back, and even grow from the pandemic to gain personal development [22–24]. These studies suggest that young people with higher levels of resilience would be more prone to experience positive changes. Besides, the buffering, bolstering, and building effects of positive psychology suggest that gaining personal growth from COVID-19 would facilitate a better mental health [14], which suggests that young people with wider (i.e., more aspects) and deeper (more intense) positive changes in life outcomes would show better mental health.

Taken together, this study examined three questions to contribute to the literature regarding adolescents’ positive changes in life outcomes over the past 2 years in a national sample of Chinese adolescents aged 12 to 24 (*N* = 2567). First, we explored different profiles of positive changes in life outcomes. This topic is exploratory in nature, but we expected that distinct profiles would emerge. Second, we examined the extent to which resilience would be related to the membership of profiles. Given the importance of resilience to successful adaptation to crises, we hypothesized that adolescents with high levels of resilience would be more likely to be in a profile indicating more positive changes. Third, we examined whether adolescents with different profiles of positive changes would have different mental health outcomes. Based on the viewpoints of positive psychology, we hypothesized that adolescents in a profile indicating more positive changes would have better mental health than those in profile indicating fewer positive changes.

## Method

### Participants and procedure

Participants of this study were from a larger survey that investigated Chinese public’s intention/motivation of getting COVID-19 vaccine and its correlates. Student helpers (*N* = 168) from various regions in China volunteered to spread an online survey link on their social networks via different social media platforms (e.g., WeChat). Within the survey period (from 23-July to 12-August, 2021), 3273 participants provided consent and completed the survey. As a quality control, we excluded a number of participants because: (1) their age was out of our expected range (< 12 or > 90 years old; *N* = 11); (2) they were living oversea (*N* = 7); (3) they had duplicate IP address (*N* = 195), or (4) they answered the survey too fast according to Leiner’s relative speed index (> 2; *N* = 75)^2^ [25]. The remaining 2985 participants constituted the final sample of the larger survey (M_age_ = 22.07 years; 1966 females).

Participants aged 12 to 24 from the said larger survey consisted of the sample of this study. The current sample size was 2567 adolescents from 32 provinces/regions in China (1717 females; M_age_ = 19.87 years, SD = 2.02). Among them, 90.8% were full-time students. In addition, 157 and 28 participants reported that they had history of physical or psychiatric illness, respectively; and 44 participants reported that they were directly related to COVID-19 (e.g., confirmed/suspicious cases and/or relatives/friends of the confirmed cases, etc.).

This study was approved by the ethical committee of Guangzhou University. The survey was conducted online. Participants (or their guardians) provided consent by checking the box in the front page of the survey that fully explained the study. They reserved the right to withdraw from the survey at any time. Participation was voluntary and no incentive was provided. We did not collect identifiable personal particulars and confidentiality was stressed.

## Measures

### Positive changes in life outcomes

We adapted the positive events subscale of the Epidemic-Pandemic Impacts Inventory used in previous study to measure Chinese adolescents’ positive changes in life outcomes [16]. The original subscale has 21 items measuring whether participants have experienced positive changes during the initial lockdown across a number of life outcomes (e.g., relationships, physical activity, sleep, work) with binary response options (i.e., yes or no) [16]. In the present study, we modified the said subscale in several aspects to suit the current participants better. First, we grouped some items that had similar meaning. For instance, in the original subscale, the items “more quality time with partner or spouse” and “more quality with children” were combined and modified as “more quality time with family members”. Second, as mentioned above, adolescents in China might have been frequently fed up with information with positive energy [18] and patriotism [26] over the past two years. In this regard, we added a few items to reflect this situation, such as “become more grateful” and “become more patriotic”. Last, we changed the binary response options to a five-point scale (from 1 = *strongly disagree* to 5 = *strongly agree*) so as to capture larger variances. A higher score indicates higher levels of positive changes in life outcomes. The final scale has 19 items. Two experts evaluated all the items, considering the items had good content validity and they were appropriate in the Chinese context. Participants were asked to rate how much they had experienced the listed positive changes in various life outcomes since the outbreak of COVID-19. The full set of items are illustrated in Table 2. The Cronbach’s α of this scale was 0.95 in this study.

### Resilience

We used the Brief Resilience Scale [27] to measure participants’ ability to bounce back. In this study, the Chinese version of BRS was used [20]. This scale has 6 items rated on a five-point scale (from 1 = *strongly disagree* to 5 = *strongly agree*). A higher mean score indicates better ability to bounce back from stressors. Sample items are “It does not take me long to recover from a stressful event” and “I usually come through difficult time with little trouble”. The Cronbach’s α of this scale was 0.69 in this study.

### Mental health

We used GHQ-12 [28] to measure participants’ mental health. The Chinese version of this scale was used [29]. This measure consists of 12 items which are either stated in negative or in positive wordings. Participants were asked to rate on these items according to their situation over the past month compared to their usual situation. All items are rated on a four-point scale (from 1 = *better than usual* to 4 = *much worse than usual*). A higher mean score indicates more mental distress (i.e., less mental health). Sample items are “feel unhappy and depressed” and “lose confidence in self”. The Cronbach’s α of this scale was 0.90 in this study.

### Covariates

We measured several demographic variables as covariates, as they had been found to be associated with various life outcomes in Chinese public at the beginning of COVID-19 [30]. These covariates include biological sex (1 = *male*, 2 = *female*), age, their relationship with COVID-19 (1 = directly related, such as confirmed/suspicious case and/or relatives/friends of the confirmed cases, etc., 2 = *not related*), history of physical and psychiatric illness (1 = *yes*, 2 = *no*), and their current physical health condition (from 1 = *very poor* to 5 = *very good*).

### Data analyses

We analyzed the data with SPSS 26.0 and Mplus 7.31 [31]. The variables examined in this study had never been used in other studies. The research questions, hypotheses, and the data analytic plan were pre-registered at aspredicted.org (protocol number: #73896). First, we conducted preliminary analyses, including means and standard deviation. Second, we carried out latent profile analysis (LPA) to examine the first research question. All the 19 items listed in Table 2 were used as the indicators of analysis. We first started with one-profile, and then increased the number of profiles systematically until we identified the best fitting model according to a number of indices, including Akaike Information Criteria (AIC) [32], Bayesian Information Criterion (BIC) [33], adjusted BIC (aBIC), Lo-Mendell-Rubin Adjusted Likelihood Ratio Test (LMRT) [34], and Bootstrapped Likelihood-Ration Test (BLRT) [35]. Smaller values of AIC, BIC, and aBIC indicated better model fit. The p-value values associated with LMRT and BLRT indicate whether the *k*-profile model (*p* < 0.05) or the *k*-1 profile model (*p* > 0.05) has a better fit. Besides, the value of entropy no less than 0.6 indicates good profile separation [36]. In addition, we also considered theoretical meaningfulness of the profile [37] and the proportion of participants represented in the profiles [38]. As a rule of thumb, no profile should have a group comprised of less than 5% of the participants [39]. Multivariate analysis of variance (MANOVA) was conducted to examine whether the mean level of each indicator significantly differed across profiles. *P*-values and effect sizes (*η*^*2*^_*p*_) were used to judge the significance. Third, we performed a logistic regression model using a three-step procedure in Mplus (i.e., R3STEP auxiliary command) [36] to examine the second research question after identifying the best-fitting model. Logits from the model output were transformed into odd ratios for explanation purposes. Demographic variables measured above were included in the model as covariates. Fourth, analysis of variance (ANOVA) was performed to examine the third research question, with the average score of GHQ as the dependent variable and the profiles as the independent variables. Both *p*-values and effect size (*η*^*2*^_*p*_) were used to determine the significance. Based on ANOVA, we further conducted ANCOVA controlling for covariates. Finally, as a robust check, we replicated the analyses for research questions 2 and 3 with winsorized scores of resilience and GHQ,^3^ respectively.

## Results

### Preliminary analysis

Descriptive analysis found that participants overall reported above-medium levels of positive changes in life outcomes (M = 3.74, SD = 0.61). Besides, participants reported medium level of ability to bounce back (M = 3.17, SD = 0.55) and relatively low levels of mental distress (M = 1.95, SD = 0.49).

### Profiles of positive changes in life outcomes

As shown in Table 1, latent profile analysis found that the 3-profile solution described the optimal number of profiles for changes in life outcomes. According to the fit indices, the 3-profile solution demonstrated a better fit than the 2-profile solution, as indicated by significant LMRT (*p* < 0.001) and BLRT (*p* < 0.001). By contrast, the 4-profile solution was not better than the 3-profile solution, as indicated by insignificant LMRT (*p* = 0.533). Besides, the values of AIC, BIC, and aBIC decreased as the number of profiles increased, and such decrease appeared more apparent between 1-profile and 2-profile solutions and between 2-profile and 3-profile solutions than the one between 3-profile and 4-profile solutions. Moreover, no group contained less than 5% of the participants for the 3-profile solution, but one group of the 4-profile solution contained less than 5% of the participants. Taken together, we selected the 3-profile solution as the final solution. This solution showed high entropy (i.e., 0.93). The average posterior profile membership probability was high, with 96.7%, 97.1%, and 97.2% for the first, second, and the third profile, respectively.

**Table 1:** Summary of latent profile models

	Log Likelihood	Number of Free Parameter	AIC	BIC	aBIC	Entropy	LMRT *p* value	BLRT *p* value	Class size per profile
1 Profile	− 61,123.07	38	122,322.13	122,544.45	122,423.71	–	–	–	2567
2 Profiles	− 53,446.73	58	107,009.47	107,348.80	107,164.51	0.92	< .001	< .001	1434/1133
**3 Profiles**	− **49,960.22**	**78**	**100,076.43**	**100,532.77**	**100,284.94**	**0.93**	** < .001**	** < .001**	**854/442/1271**
4 Profiles	− 48,553.91	98	97,303.82	97,877.16	97,565.79	0.95	.533	< .001	62/413/932/1160

Bolded entries represent the solution chosen in this study

Table 2 presents the raw score of each indicator in each profile and the results of MANOVA. We labelled the first profile as “*limited positive changes*”. This profile consisted of 33.3% of participants. Participants in this profile showed positive changes in only a few aspects of life outcomes, mainly concerning personal health (e.g., item 3 & 14) and social responsibility (e.g., items 13 & 15), and such changes were moderate, as their scores were not far from the mid-point (i.e., 3.00). We labelled the second profile as “*overall strong positive changes*”. This profile contained about 17.2% of the participants. Participants in this profile reported positive changes in all the listed aspects of life outcomes at high level (i.e., > 4.00). We labelled the third profile as “*partial positive changes*”. This profile contained about half of the participants (49.5%). Participants in this profile showed positive changes in several aspects related to self-care (e.g., items 4, 5, & 14), positive virtues (e.g., items 7 & 12), social responsibility (e.g., items 11, 13 & 15), and emotional and psychological well-being (e.g., items 17 to 19). The magnitudes of changes in these aspects were higher than the mid-point but did not reach high levels (> 4.00).

**Table 2:** Means, standard deviations, and MANOVA tests for indicators among the three profiles

Items	Profile 1		Profile 2		Profile 3		*F*(2, 2564)	*p*	*η*^*2*^_*p*_
	M	SD	M	SD	M	SD			
1.More quality time with family members	3.03	0.78	4.51	0.73	3.53	0.78	533.63	< .001	.29
2.Improved relationships with family	2.90	0.66	4.44	0.77	3.39	0.71	699.64	< .001	.35
3.Improved relationships with friends	2.94	0.62	4.40	0.83	3.34	0.69	653.81	< .001	.34
4.Pay more attention to personal physical health	3.50	0.76	4.81	0.46	4.10	0.60	632.62	< .001	.33
5.Have more time to explore and cultivate new hobbies	3.03	0.73	4.62	0.64	3.63	0.73	725.88	< .001	.36
6.Have better quality of sleep	2.71	0.70	4.29	0.94	3.12	0.76	609.41	< .001	.32
7.Become more grateful	3.14	0.71	4.78	0.44	3.92	0.61	1052.79	< .001	.45
8.Live a healthier life (e.g., healthier diet, less smoking & drinking)	3.04	0.71	4.79	0.48	3.83	0.64	1122.81	< .001	.47
9.Spent less time on screens or devices outsides of work/study hours	2.46	0.79	4.13	1.09	2.94	0.87	518.70	< .001	.29
10.Be more appreciative of your current work/study	2.96	0.67	4.70	0.62	3.72	0.63	1094.71	< .001	.46
11.Become more helpful (e.g., donate goods, do voluntary work)	3.21	0.64	4.81	0.45	3.95	0.55	1193.33	< .001	.48
12.Become more respectful to others	3.21	0.61	4.83	0.42	4.01	0.51	1413.58	< .001	.52
13.Become more concerned about national and international news	3.70	0.73	4.91	0.33	4.29	0.54	657.61	< .001	.34
14.Cherish the life even more	3.64	0.76	4.94	0.29	4.40	0.52	803.40	< .001	.39
15.Become more patriotic	3.84	0.81	4.96	0.19	4.50	0.53	570.77	< .001	.31
16.Found greater meaning in your own life	3.08	0.64	4.89	0.34	4.01	0.61	1492.07	< .001	.54
17.Experience more positive emotions (e.g., happy, joyful, excited)	2.90	0.60	4.88	0.33	3.87	0.65	1710.68	< .001	.57
18.Become more optimistic about the future	2.94	0.60	4.90	0.31	3.98	0.60	1917.75	< .001	.60
19.Feel stronger well-being	2.97	0.61	4.89	0.32	3.95	0.63	1697.65	< .001	.57

We conducted a MANOVA analysis to examine whether the indicator scores were different across profiles. The results indicated a significant multivariate test, Wilk's λ = 0.17, *F*(38, 5092) = 188.61, *p* < 0.001, *η*^*2*^_*p*_ = 0.59. Results of between-subject effects revealed significant main effect for each indicator. Post-hoc multiple comparison with Bonferroni correction found that the *over strong positive changes* profile had significantly higher levels on all indicators than the other two profiles and that the *partial positive changes* profile had significantly higher levels on all indicators than the *limited positive changes* profile.

### The association between resilience and different profiles

The results of logistic regression are summarized in Table 3. As shown, compared to the *limited positive changes* profile, participants with 1 unit increase in resilience were 2.01 and 1.43 times more likely to be the members of the *overall strong positive changes* and the *partial positive changes* profile, respectively, net the effects of multiple covariates. Moreover, compared to the *partial positive changes* profile, participants with 1 unit increase in resilience were 1.40 times more likely to be the members of the *overall strong positive changes* profile, even after controlling for covariates. In addition, replication with winsorized score of resilience as the predictor yielded very similar findings.

**Table 3:** Logistic regression of class membership on predictors

Predictors	*Estimate*	*SE*	*p*	*OR*	*OR 95% CI*
Profile 1 (reference group) vs. Profile 2					
Resilience	0.70	0.13	< .001	2.01	[1.56, 2.60]
Sex (1 = male, 2 = female)	− 0.05	0.13	.698	0.95	[0.73, 1.23]
Age	− 0.13	0.03	< .001	0.88	[0.83, 0.93]
Relation with COVID-19 (1 = related, 2 = non-related)	0.91	0.53	.089	2.48	[0.88, 7.02]
History of physical illness (1 = yes, 2 = no)	0.51	0.34	.132	1.67	[0.86, 3.24]
History of psychiatric illness (1 = yes, 2 = no)	− 0.41	0.68	.552	0.66	[0.18, 2.52]
Current physical health status	0.84	0.10	< .001	2.32	[1.90, 2.82]
Profile 1 (reference group) vs. Profile 3					
Resilience	0.36	0.10	< .001	1.43	[1.18, 1.74]
Sex (1 = male, 2 = female)	0.26	0.11	.012	1.30	[1.05, 1.61]
Age	0.01	0.02	.708	1.01	[0.97, 1.05]
Relation with COVID-19 (1 = related, 2 = non-related)	0.82	0.36	.020	2.27	[1.12, 4.60]
History of physical illness (1 = yes, 2 = no)	0.13	0.19	.513	1.14	[0.78, 1.65]
History of psychiatric illness (1 = yes, 2 = no)	0.20	0.50	.694	1.22	[0.46, 3.25]
Current physical health status	0.49	0.07	< .001	1.63	[1.42, 1.87]
Profile 3 (reference group) vs. Profile 2					
Resilience	0.34	0.11	.003	1.40	[1.13, 1.74]
Sex (1 = male, 2 = female)	− 0.32	0.12	.011	0.73	[0.57, 0.92]
Age	− 0.13	0.03	< .001	0.88	[0.83, 0.93]
Relation with COVID-19 (1 = related, 2 = non-related)	0.09	0.55	.873	1.09	[0.37, 3.22]
History of physical illness (1 = yes, 2 = no)	0.39	0.34	.253	1.48	[0.76, 2.88]
History of psychiatric illness (1 = yes, 2 = no)	− 0.61	0.65	.350	0.54	[0.15, 1.94]
Current physical health status	0.36	0.09	< .001	1.43	[1.20, 1.71]

### The differences in mental health among different profiles

The results of ANOVA analysis found a significant main effect across the *limited positive changes* (M = 2.09, SD = 0.45), *partial positive changes* (M = 1.91, SD = 0.44) and *overall strong positive changes* (M = 1.79, SD = 0.62) profiles, *F*(2, 2564) = 65.77, *p* < 0.001, *η*^*2*^_*p*_ = 0.05. Post-hoc multiple comparison with Bonferroni correction found that the adolescents in the *overall strong positive changes* profile had a significantly lower level of mental health problems than those in the other two profiles and that adolescents in the *partial positive changes* profile had a significantly lower level of mental health problems than those in the *limited positive changes* profile. We further conducted ANCOVA analysis controlling for covariates, the effect of profile on mental health problems remained significant, *F*(2, 2558) = 32.10, *p* < 0.001, *η*^*2*^_*p*_ = 0.02. In addition, replications with winsorized score of GHQ-12 as the outcome yielded very similar findings for both ANOVA and ANCOVA analyses.

## Discussion

According to the two continua model of mental health and ideas from positive psychology [11, 13], young people may also experience positive changes when they are faced with uncertainty and adversity. However, the existing literature has disproportionately focused on adolescents’ mental distress and surprisingly little research has investigated the extent to which young people would experience positive changes in times of COVID-19 pandemic. This study bridged this gap with three findings. First, we revealed three qualitatively distinct profiles of positive changes in life outcomes in Chinese adolescents. Second, adolescents with a higher level of resilience were more likely to be in a profile indicating more positive changes. Third, more positive changes in life outcomes were related to better mental health.

Previous studies found that while some life outcomes (e.g., screen time, mental health, and well-being) deteriorated during COVID-19, other outcomes (e.g., psychosomatic health and sleep time) might improve [8, 9]. The emergence of three different profiles of this study reconciles the said inconsistent findings. A recent review has pointed out that people’s adjustment outcomes may vary across domains and over time during COVID-19, with some people showing better outcomes in some aspects at a certain period and other people in other aspects at a different period [20]. Our findings are consistent with this viewpoint. As illustrated in Table 2, some participants showed positive changes in more aspects and reported higher levels of positive changes than others. This finding is also consistent with prior research that found that Scottish public generally reported positive changes in multiple life domains but that the intensity of changes varied across domains [16].

Of note, participants across profiles showed relatively higher positive changes in life outcomes related to personal health and survival (e.g., pay more attention to physical health) and to social and ideological issues (e.g., become more patriotic). According to terror management theory [40], the former may reflect that adolescents manage the anxiety induced by the pandemic through actively engaging in thoughts and behaviors that prolong their survival. The latter may result from the (positive) information spread among the Chinese public during COVID-19 [18]. Of note, a crucial reason why adolescents reported more positive changes in patriotism would be related to Chinese government’s strong intervention on the internet, such that the government use social media to distribute positive energy while discouraging any contents related to “negative energy” [18, 19]. As young people aged 10–29 are main users of internet and social media in China (China Internet Network Information Center, 2021) [41], their ideology is more likely to be affected by social media contents. In addition, schools of different levels are important places for youth patriotism education in mainland China [42], suggesting that students would be more likely to have such positive changes compared to non-students. However, given that we did not focus specially on patriotism in this study and the number of non-student participants was less than 10% of the sample size, we did not further compare whether patriotism of students would be higher than that of non-students, and this would require further research in the future as appropriate.

Moreover, participants across profiles showed relatively lower levels of positive changes in issues related to routines (e.g., spent less time on screen and sleep quality). This may be because school closure has been enforced and online learning via digital devices has been adopted from time to time in different regions over the past two years. Regarding screen time, research found increased in frequency and duration of recreational internet use in Chinese children and adolescents, especially among boys [43], probably because of more access to digital device during the pandemic. In sum, these findings implied that the preventive measures appeared to affect Chinese adolescents’ routine quite extensively.

The age range that defines adolescence may affect the current findings and this deserves more discussion. Sawyer et al. defined adolescence as young people aged 10 to 24 years considering multiple aspects, such as brain development, completion of higher education, marriage, parenthood, and adult responsibility [3]. Such definition aligns with the Chinese society where more than half of young people are now receiving tertiary education right after high school, completing their college/university at their mid-20 s, and postponing the timing for marriage and parenthood [44, 45]. As such, using Saywer et al.’s age range to define adolescence appears acceptable in the current study. Nevertheless, adolescents with different ages may be affected by COVID-19 differentially, which may therefore affect the formation of profiles. For instance, the COVID-19 may affect school-going adolescents (who are usually younger) more on school-related issues, such as disruption of school routines while the pandemic may affect working adolescents (who are usually older) more on the issues related to (un)employment and financial stress [46]. In addition, our current results showed that younger adolescents experienced fewer positive changes in life outcomes. This may be because older adolescents (e.g., college/university students) have better cognitive ability and more wisdom to evaluate and appreciate the governments and people’s efforts in fighting against the pandemic. As such, they have experienced more positive changes. However, we must acknowledge that school-going adolescents were over representative and most of them were college or university students. Given this, we were unable to further examine whether adolescents at different ages/school levels would form different profiles. Thus, we encourage future studies to re-examine this issue among adolescents of different ages and with different demographic status (e.g., students vs. non-students).

As predicted, adolescents with a higher level of resilience were more likely to be in the profile indicating more positive changes. According to the risk-resilience model, individuals with more personal or social assets would better cope with stressors to maintain adjustment [47]. In support of this idea and prior findings [22, 27], the current results suggest that resilience is an essential personal asset that helps adolescents navigate the stressors during COVID-19 and achieve more positive changes. One possible explanation may be because resilience supports adolescents’ reflective functioning, which is an essential factor that facilitates effective meaning-making from stressful events and personal growth [48–50]. Of note, individual’s ability to reflect and make meaning depends on their wisdom and cognitive ability which also increase as they age [51]. This, again, suggests that age may have implications on the current findings. Future research may also re-examine the role of resilience/meaning-making ability in the experience of positive changes in life among youth of different ages. A clinical implication of the finding is that enhancing adolescents’ ability to bounce back might be a promising way to help them achieve personal growth during the pandemic.

Adolescents perceiving overall strong positive changes in multiple life outcomes over the past two years showed better mental health over the past month, which confirmed our hypotheses. This finding is not surprising, as participants who are resilient, successfully recover from, and effectively make meaning of the pandemic are more likely to have better adjustment, such as mental health [20]. Nevertheless, what merits attention is that the levels of mental health problems found in this study were relatively low, in contrast to prior findings that revealed adolescents, both in China and worldwide, showed salient mental health problems during COVID-19 [52–56]. One possible reason to explain such difference is that most of the prior findings were conducted at the first and second stages of COVID-19 while the current research was conducted at the third stage of COVID-19 in China. According to Fegert et al.’s (2020) three-stage model of pandemic, people’s mental health is supposed to be more disrupted at stages one (i.e., when the pandemic starts) and two (i.e., when the pandemic peaks), but people’s mental health is supposed to proliferate as COVID-19 enters the last stage (i.e., back to normality). By the time of data collection, the Chinese government has well controlled the pandemic and people’s lives has largely returned to normality. As such, it is not surprising that our participants showed relatively low levels of mental distress.

This research has several limitations. First, the life outcomes examined in this research are not exhausted, although these outcomes are appropriate for Chinese adolescents and most of them have been examined in prior research [16]. Second, this sample was not nationally representative, as girls were over represented according to Chinese census [57] and the sample size of different regions differed considerably. Third, utilizing longitudinal design to examine adjustment during COVID-19 is promising as it allows direct comparison across periods [20], but the design of this research is cross-sectional in nature. Nevertheless, we consider that the current findings are still valuable in that they reflect adolescents’ *overall perception* of the positive changes in life outcomes over the past two years. These results are meaningful, as scant research has specifically examined this issue so far. Future research may continue examining this topic with standardized measures, more representative samples, and longitudinal design. In addition, cross-cultural comparison would be also promising, as how adolescents perceive their lives have changed is supposed to be related to the pandemic situation surrounding them and the effectiveness/side effects of the measures adopted in different nations/regions.

## Conclusion

Despite the negative psychosocial effects induced by COVID-19 on adolescent development, it is also necessary to understand young people’s positive changes, which is important to shed light on personal growth in times of crisis. Our research finds that Chinese adolescents report positive changes in various life outcomes at different levels, especially for resilient adolescents. Such changes have important implications on young people’s mental health. Given that the current topic is crucial yet largely underexplored, we encourage future research to focus more on adolescents’ positive development in times of the COVID-19 pandemic.

## Data Availability

The measures are attached as supplementary files and the dataset is available from the corresponding authors upon request.

## Footnotes

1 We pre-registered that this study would focus on adolescents aged 12 to 25. But in this study we decided to focus on adolescents aged 12 to 24 to follow the definition of adolescents by Sawyer et al. (2018).

2 Leiner’s relative speed index was calculated in the following steps. First, although the questionnaires administered to participants who had and who had not got vaccinated were largely the same, several questionnaires were different, thus resulting in different completion time between the two groups of participants. Hence, we split the two groups and gauged the relative speed index of each participant for each group. Second, we calculated the median completion time for each group. Third, we used each participant’s completion time to divide the median completion time of the group he/she was in, and a relative speed index could be obtained. For instance, if the median completion time of the non-vaccinated group was 600 s, a participant in this particular group had a completion time of 200 s. Then, this participant’s relative speed index was 600/200 = 3 and he/she should be excluded because his/her index was larger than the cut-off point (i.e., 2).

3 Winsoring approach (Tukey, 1962) was used to handle the outliers of the resilience and GHQ by replacing the outliers with the nearest number within the -3 to + 3 SD range.
